# PTSD, FOMO and fake news beliefs: a cross-sectional study of Wenchuan earthquake survivors

**DOI:** 10.1186/s12889-023-17151-z

**Published:** 2023-11-09

**Authors:** Chen Gong, Yijin Ren

**Affiliations:** 1 School of Journalism, Fudan University, No. 440, Handan Road, Shanghai, 200433 China; 2 Mianyang College of Administration, Sichuan, China

**Keywords:** Wenchuan Earthquake, Fake news, Post-traumatic stress disorder, Fear of missing out, Social Media Use

## Abstract

**Background:**

Post-traumatic stress disorder (PTSD) sufferers show problematic patterns of Internet use such as fear of missing out (FOMO) and sharing misinformation and fake news. This study aimed to investigate these associations in survivors of the 2008 earthquake in Wenchuan, China.

**Methods:**

A self-reported survey was completed by 356 survivors of the Wenchuan earthquake. A mediated structural equation model was constructed to test a proposed pattern of associations with FOMO as a mediator of the relationship between PTSD symptoms and belief in fake news, as well as moderators of this pathway.

**Results:**

PTSD was directly associated with believing fake news (β = 0.444, p < .001) and with FOMO (β = 0.347, p < .001). FOMO mediated the association between PTSD and fake news belief (β = 0.373, p < .001). Age moderated the direct (β = 0.148, t = 3.097, p = .002) and indirect (β = 0.145, t = 3.122, p = .002) pathways, with effects more pronounced with increasing age. Gender was also a moderator, with the indirect effect present in females but not in males (β = 0.281, t = 6.737, p < .001).

**Conclusion:**

Those with higher PTSD symptoms are more likely to believe fake news and this is partly explained by FOMO. This effect is present in females and not males and is stronger in older people. Findings extend knowledge of the role of psychological variables in problematic Internet use among those with PTSD.

On May 12, 2008, a magnitude 8.0 earthquake struck Wenchuan County, Sichuan Province, China [1], causing hundreds of thousands of fatalities and $150 billion in damages [1, 2]. The Wenchuan earthquake was a catastrophic event that prompted a great deal of research. According to Fan et al. [2], approximately 7,000 peer-reviewed articles about the earthquake had been published by the end of 2018. Notably, however, few of these articles documented the PTSD experienced by earthquake survivors.

PTSD is proved that it could significantly contribute to online information engagement, which indicates the increased exposure to Internet disinformation, as investigated the relationship between PTSD and disinformation engagement among adolescents who have experienced a major earthquake [3, 4]. The effects of PTSD are pervasive and far-reaching. PTSD is linked to problematic smart device use, which some academics have termed ‘iDisorder’ [3]. A recent study of adolescent earthquake survivors found that PTSD may also contribute to online disinformation exposure and engagement [4]. Several studies have been conducted to explore the reasons for these links, most with a focus on younger PTSD sufferers [4–6].

Researchers have explored relationships between PTSD and other aspects of maladaptive technology use [5]. One such aspect is the fear of missing out, or FOMO, which is characterized by a persistent need to stay connected to the experiences of others, typically through using social media [7–9]. FOMO is a widespread phenomenon that can be detrimental to physical and mental health [10]. It is not necessarily bad, as it can enhance the strength of social media connections and improve happiness in some [11]. In most people, however, it seems to be associated with a maladaptive ruminative style of thinking that can cause negative outcomes such as increased anxiety. Rumination refers to the vicious cycle that occurs when individuals repeatedly recall their feelings after a stressful life event. Studies have found that women and single people show higher levels of FOMO and ruminative thinking than men and married people [12, 13].

FOMO is also linked with the intensity of problematic smartphone use to assess their mediating role in PTSD [3, 14–16], and the results indicated that problematic smartphone use was most strongly associated with negative emotions and arousal in individuals with PTSD [14]. FOMO is a mental health-related construct that may drive some negative emotion, but PTSD remains the dominant concept because it can drive both excessive Internet usage and misinformation.

And with social media tools providing an ever-increasing amount of social information, there is a close proximity to real-time media activity on various social networks. This digital activity-driven flood of updates has given rise to a relatively new phenomenon known as FOMO [9, 11, 17]. Excessive social media use has been shown to cause significant fatigue, which is followed by high anxiety and depression, and FOMO plays an indirect role to predict this [18]. Przybylski attributes this phenomenon to the fact that social platforms offer benefits to the general public, the internet is likely a particularly effective tool for combating FOMO. In fact, media news provide a ‘efficient and low friction path’ [9], particularly for those who follow the news constantly. FOMO has also been studied in relation to news information, business information engagement and fake news [12]. The spread of nonexistent fake news on social media poses a serious threat to the vast majority of social platforms. In 2016, for instance, the sports brand New Balance faced widespread backlash from anti-Trump groups after rumors circulated that its products had been misbranded as being associated with the then-Trump presidential campaign. This resulted in a decline in the company’s stock price. In the same year, there was a massive Pepsi boycott after senior Pepsi employees were arrested for showing government hostility. Companies, governments, and even individuals can generate and disseminate information (or news) in order to quickly deliver their own agendas to large audiences via social media, making the threat of fake news imminent [17]. Few studies, however, have examined the relationship between fake news and FOMO, and fake news videos have begun to emerge, which, due to their novelty and potent visual impact, increase the risk of anxiety, and FOMO was found to be a direct contributor to this situation [19]. In contrast, based on responses from China and the United States, Jo et al. [20] investigated the mediating relationship between FOMO and misinformation trust across multiple age and cultural contexts. The findings show that older individuals are especially susceptible to misinformation because they are less likely to check suspicious content and have less motivation to share information online, so the age is also an important moderator variable in the study of the relationship between FOMO and misinformation. However, FOMO is not always a bad thing, as it has a positive effect on happiness and indirectly affects the strength of social media connections [11], despite the fact that other studies of FOMO’s relationship to happiness have primarily negative attitudes. For instance, Hattingh et al. [21] examines the association between FOMO and misinformation on social media, which is mediated by information and communication overload, online subjective well-being, whereas Leung et al. [22] examined the mechanisms of disinformation spread and depressive symptoms through three studies with the aim of highlighting the mediating role of FOMO.

Therefore, it may be essential to comprehend how FOMO and negative emotions (such as depression, anxiety, stress disorders, etc.) contribute to the varying levels of misinformation belief among the general public. By building up a theoretical framework for PTSD, FOMO, and fake news beliefs, this study will fill a gap in the literature by examining Wenchuan earthquake survivors to determine if their PTSD symptoms may contribute to their belief in fake news via the mediating effect of FOMO.

## Review and hypothesis

### The association between PTSD and fake news belief

Though research into fake news has been conducted in many academic fields, we focus here on belief in fake news and the psychological variables associated with such belief, such as negative emotions, anxiety, depression, and PTSD symptoms. As yet, few studies have investigated the link between negative emotions and engagement with so-called ‘dark phenomena’ on social media [21]. Delmastro and Paciello [23] suppose that the relationship between PTSD and trust in disinformation is a complex one, while Yang et al. [4] found a significant link between PTSD and exposure to Internet misinformation. The authors also highlight the role of news recommendation algorithms in social platforms, as algorithms can lead to echo chambers and confirmation bias for users, which is a major issue in believing misinformation and fake news [23]. They also discovered that people living alone were significantly more likely to believe and retweet fake news, and physical isolation in the real world (such as natural disasters, epidemics, etc.) may contribute to the belief in fake news.

Studies have revealed that psychological problems linked to infectious disease outbreaks may increase trust and retweeting of fake news on social platforms and that PTSD may be linked to dependence on social media platforms [4]. Other individual factors linked to trust in fake news include level of education, with better-educated individuals more able to discern the accuracy of information, and physical isolation, with individuals who live alone being more likely to believe and retweet fake news [24].

### The mediating effect of FOMO

Numerous scholars have examined cognitive-psychological factors including FOMO as correlates of belief in fake news [11, 17–19, 22, 25, 26]. Talwar et al. [25], for example, found that FOMO, along with online self-disclosure and social media fatigue, were positively associated with sharing fake news. FOMO is skewed towards negative outcomes, which also explains depressive symptoms and PTSD respectively [17]. Another study [11] explains that FOMO is positively related to the intensity of online disinformation exposure and negatively related to social connections.

In some cases of social isolation, people may experience frequent FOMO when they are away from their social environment because they are not seeing their friends, partners, or family members [13]. It is reasonable to assume that the destruction of social networks caused by the earthquake is also a cause of FOMO. Other studies on the relationship between PTSD and FOMO typically include mediator variables, such as problematic mobile phone use [3, 8, 16, 27], anxiety or depression [5, 6, 10, 15, 16, 28], compensatory internet use [15], etc., and also consider the role of certain moderator variables, such as family environment [6], adulthood or not [4, 6]. Compensatory Internet use theory posits that FOMO and sharing false information may be markers of problematic Internet use that is engaged in to escape from real-world problems or reduce dysphoric moods [12].

In addition, FOMO usually plays as a mediator variable to expand PTSD and the domain of psychopathology-related constructs. Elhai et al. [7] was one of the first to examine the relationship between FOMO and PTSD severity in Asian subjects, and they discovered that FOMO may be an important variable in explaining certain types of psychopathology. In another study by Elhai et al. [8], he expressed concern that few studies had examined mental health variables associated with smartphone use and misinformation sharing.

Through a literature review, Tandon et al. [17] identified the limitation of all FOMO articles, namely that previous studies have focused on settings where the FOMO variable is associated with social media related concepts. The authors suggest continuing to validate the relationship between FOMO and fake news beliefs in settings that are disconnected from real-world scenarios. Social media news use and FOMO were positively correlated with intentional deepfake sharing, suggesting that individuals with lower cognitive abilities presented higher levels of FOMO and more sharing behaviour [19], which demonstrated that the indirect effect of social media news sharing on the progression of FOMO is greater for low cognitive individuals, and reveals for the first time that even those with a higher analytical mindset are susceptible to fake news, particularly deepfakes. In accordance with the above literature review, we therefore propose the following research hypothesis:

### The moderating effect of age and gender

There is some evidence that any relationship that exists between FOMO and susceptibility to misinformation is dependent on demographic and cultural variables including gender [5, 29], family environment [6], and age [4, 6]. In a study of participants from China and the United States, Jo et al. [20] found older individuals to be especially vulnerable to misinformation because they are less likely to fact-check suspicious content. Elhai et al. [7] demonstrated associations between FOMO and demographic characteristics including age, gender, ethnicity, and relationship status. In another study, Elhai [29] and colleagues studied the relationship between FOMO and sharing fake news sharing on social platforms, finding that FOMO increased sharing of fake news and that age moderated this relationship, with the link stronger in older respondents than younger ones. Alt [26] examined FOMO and motivation for sharing fake news in students, finding that millennials of ethnic minorities were subject to pressure from their ethnic group which increased the likelihood of them spreading fake news. In another study, cognitive ability moderated a correlation between FOMO and intentional deepfake sharing, suggesting that individuals with lower cognitive ability had higher FOMO and were more likely to share deepfakes [19].

Some studies have confirmed gender differences in the prevalence of FOMO, such that it is higher among females [30]. In one study [5], the authors interpreted this by linking females’ higher incidence of depression with use of social networking sites, while in another, FOMO was linked to depression in females [16]. Another consideration is that generally more women than men participate in misinformation studies [4, 24], meaning that moderation effects in these studies could be an artefact of unbalanced gender distribution.

### Aims and hypotheses

The main purpose of this study is to establish the structure of associations between PTSD, FOMO, and misinformation belief in survivors of the Wenchuan earthquake. We are also interested in exploring moderators of these associations. Based on the above review of relevant literature, we propose the following seven hypotheses for the current study:

#### H1

PTSD caused by the Wenchuan earthquake is positively associated with FOMO.

#### H2

FOMO is positively associated with believing fake news.

#### H3

PTSD is positively associated with believing fake news.

#### H4

FOMO mediates the association between PTSD and believing fake news.

#### H5

Age moderates the PTSD–fake news pathway.

#### H6

Age moderates the FOMO–fake news pathway.

#### H7

Gender moderates the FOMO–fake news pathway.

**Fig. 1:**
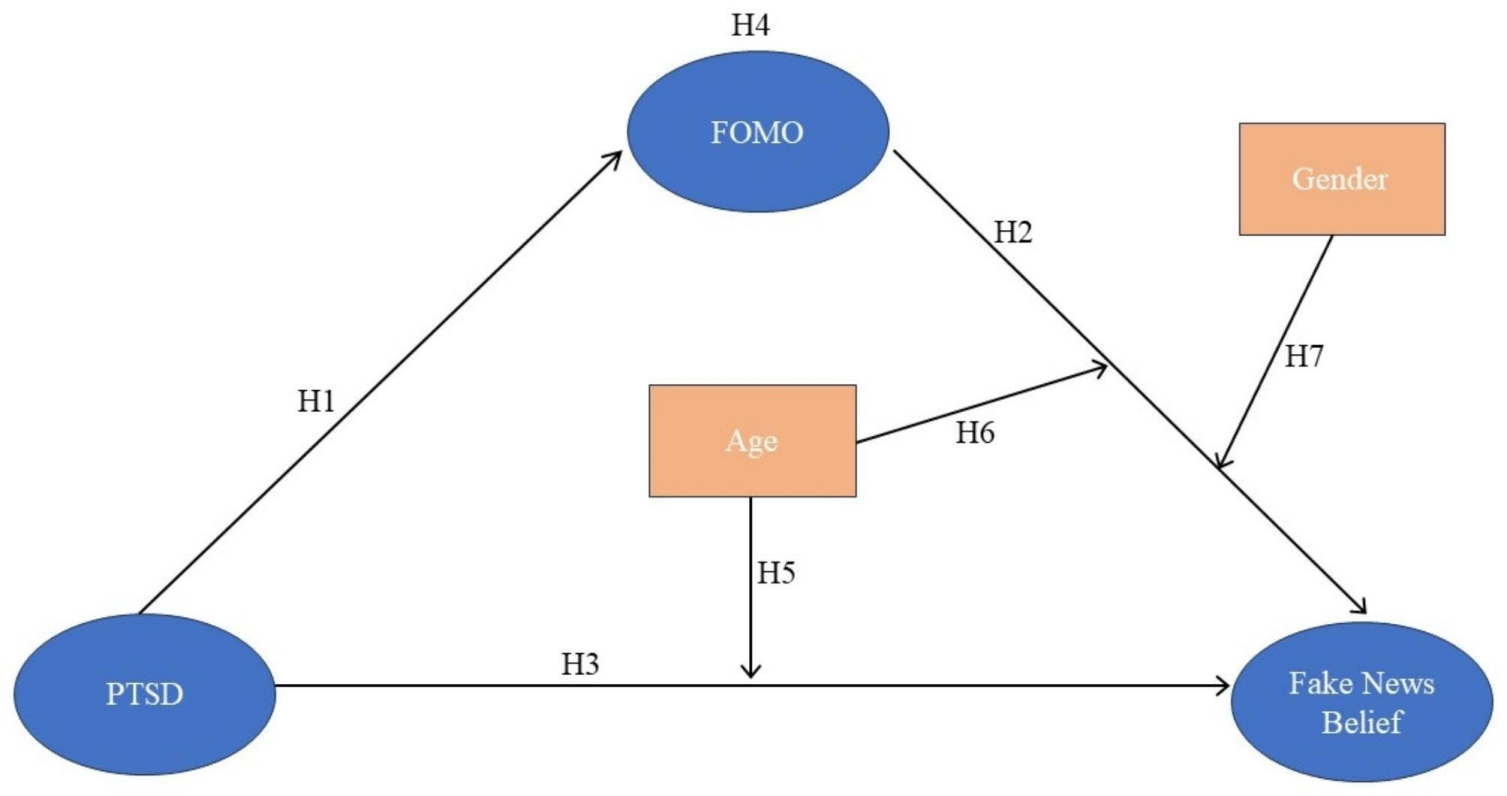
Research model

Figure 1 presents a model visualising these hypothesized relationships.

## Method

### Participants and procedure

From January to March 2023, we mailed and visited earthquake survivors in Beichuan County, a particularly hard-hit area of the Wenchuan earthquake. Inclusion criteria were that participants had normal hearing and vision, no concussion in the week before the test, and did not use psychostimulants or other substances that could affect the central nervous system. We distributed 800 copies of our survey. Once returned, we eliminated those with missing data and irregular responses, leaving a total of 356 valid copies and an effective response rate of 44.5%. Our final sample included 180 men and 176 women aged between 20 and 47 (30.42 ± 5.41).

### Ethics

The study was approved by the Ethics Committee of Mianyang College of Administration. All participants gave written informed consent and were compensated for their time.

### Measures

#### PTSD Scale

The majority of published research on PTSD has been conducted in a Western context. As such, there is relatively limited knowledge of PTSD in the Chinese population. As Leung et al. (2021) note, however, cultural context is an important factor to consider. We therefore referred to previous peer-reviewed studies that have used relevant measures translated for use with Chinese participants [20, 31]. One commonly used measure is the adult version of the PTSD Checklist - Civilian Version [32], and its subsequent modified versions [31], which show good validity and reliability in Chinese samples. The PTSD scale used here consists of 17 items, each with a five-point Likert response scale ranging from 1 = strongly disagree to 5 = strongly agree. An example item is “*Even if nothing reminds you of it, you remember or have mental images of the traumatic event*”. Higher total scores indicate more severe PTSD. Reliability analysis showed a Cronbach’s α of 0.952. Specific Cronbach’s α values of each PTSD scale item and other indicators are available at https://osf.io/3v9t8/.

#### FOMO Scale

Based on previous studies with Chinese participants [7, 20, 22, 31], we used Przybylski’s [9] scale to form a Chinese FOMO scale. Responses were given on a 5-point scale ranging from 1 = not at all true of me to 5 = extremely true of me. An example item is “*I fear others have more rewarding experiences than me*”. Higher total scores indicate more severe experience of FOMO. Cronbach’s α was 0.943 in this sample.

#### Fake news belief Scale

We developed a five-item scale based on previous studies of social media use [4, 19, 33, 34] Responses were given on a 5-point scale ranging from 1 = not at all true of me to 5 = extremely true of me. An example item is “*Even if the news is not published by official media, you focus more on the news’s content than on its publisher*”. Higher total scores indicate stronger belief of fake news. Cronbach’s α was 0.864.

## Results

### Factors analysis

Reliability analysis revealed that Cronbach’s α coefficients ranged from 0.864 to 0.952. As values greater than 0.7 are considered to be good [35], the scales used here had excellent reliability.

To account for possible common method bias from using only self-reported questionnaires [36], we conducted a Confirmatory Factor Analysis (CFA) using AMOS 24 software. In CFA, factor loadings of individual items must be above 0.6 and model fit must meet appropriate criteria. Structural equation model fit metrics are used for cardinality, degrees of freedom, cardinality/degrees of freedom, RMSEA (Root Mean Square Error of Approximation), GFI (Goodness of Fit Index), AGFI (Adjusted Goodness of Fit Index), and RFI (Relative Fit Index), NFI (Normed Fit Index), IFI (Incremental Fit Index), TLI (Tucker-Lewis index) and CFI (Comparative Fit Index). We refer to the format of the Table 2 result reported by Li et al. [37] and form the analysis in Table 1. As shown in Table 1, all model fit indicators fell within a reasonable range, and the model fit the data well indicating our data are suitable for further analysis.

We also followed recommendations from Hair [38] regarding standardized factor loadings, which state that convergent validity can be assumed if composite reliability (CR) is over 0.7 and average variance extracted (AVE) is over 0.5. Analysis showed standardised factor loadings for each item ranged from 0.699 to 0.804, with all exceeding 0.6 and the significance threshold. CR values ranged from 0.864 to 0.952 and AVE values ranged from 0.540 to 0.563. Therefore, the data showed good convergent validity and passed the validation factor analysis.

**Table 1:** Confirmatory factor analysis

Factor	Items	Coef.	se	Z	Std. estimate		CR	AVE	Fit metrics
PTSD	A1	0.600	0.039	15.288	0.713		0.952	0.540	χ2/df = 1.06 RMSEA = 0.013 GFI = 0.916 AGFI = 0.905 RFI = 0.921 NFI = 0.926 IFI = 0.995 TLI = 0.995 CFI = 0.995
	A2	0.721	0.044	16.479	0.753				
	A3	0.671	0.042	15.893	0.733				
	A4	0.655	0.043	15.177	0.709				
	A5	0.624	0.042	14.941	0.701				
	A6	0.757	0.044	17.232	0.776				
	A7	0.725	0.044	16.378	0.749				
	A8	0.660	0.042	15.757	0.729				
	A9	0.643	0.040	16.177	0.743				
	A10	0.586	0.039	14.874	0.699				
	A11	0.680	0.043	16.027	0.738				
	A12	0.756	0.046	16.333	0.748				
	A13	0.716	0.044	16.240	0.745				
	A14	0.593	0.039	15.093	0.706				
	A15	0.633	0.040	15.919	0.734				
	A16	0.614	0.038	16.211	0.744				
	A17	0.635	0.038	16.892	0.766				
FOMO	B1	0.754	0.049	15.379	0.718		0.944	0.563	
	B2	0.832	0.050	16.742	0.763				
	B3	0.780	0.048	16.280	0.748				
	B4	0.688	0.045	15.307	0.715				
	B5	0.848	0.051	16.532	0.756				
	B6	0.802	0.051	15.894	0.735				
	B7	0.775	0.049	15.872	0.734				
	B8	0.797	0.051	15.537	0.723				
	B9	0.905	0.052	17.589	0.789				
	B10	0.741	0.045	16.643	0.760				
	B11	0.812	0.051	16.031	0.740				
	B12	0.904	0.050	18.096	0.804				
	B13	0.837	0.051	16.383	0.751				
Fake News Beliefs	C1	0.483	0.031	15.579	0.744		0.864	0.561	
	C2	0.508	0.033	15.437	0.740				
	C3	0.479	0.031	15.400	0.738				
	C4	0.494	0.029	16.971	0.791				
	C5	0.469	0.031	15.168	0.730				

### Descriptive statistics and correlations

Descriptive statistics for the principal scales and Bivariate Pearson correlations between the scale are shown in Table 2. PTSD prevalence was 38.1%, which is consistent with Wang et al. [39] and Ying et al. [40], with 28.4% and 42.5% respectively. Given the fact that this study is not a PTSD population survey, and the amount of people surveyed was not as large as in the two Wenchuan earthquake related PTSD studies of Wang et al. [39] and Ying et al. [40], and the area surveyed was not expansive as them, so the rate presented in this study needs to be further investigated. And the results show that PTSD was significantly correlated with FOMO (r = .347, p < .01; H2), and Fake News Belief (r = .444, p < .01; H3), with effect sizes ranging from moderate to large. And FOMO is also correlated with Fake News Belief (r = .482, p < .01; H1).

**Table 2:** Descriptive statistics and correlations

Scale	M	SD	Gender	Age	PTSD	FOMO	Fake News Belief
Gender	0.494	0.501	1.000				
Age	30.424	5.412	0.061	1.000			
PTSD	52.806	11.563	-0.003	-0.044	1.000		
FOMO	39.379	10.800	-0.069	0.036	0.347^**^	1.000	
Fake News Belief	9.927	2.621	-0.037	-0.028	0.444^**^	0.482^**^	1.000

Note: Gender, 0 = male, 1 = female;**p < .01, two-tailed test.

### Regression and mediation analyses

To test for mediation, we first employed a regression analysis using the three-step method [38] followed by an additional 5,000 simulations using bootstrapping to calculate confidence intervals for indirect effect bias correction. If the confidence interval does not contain zero, the indirect effect holds.

Analysis showed that PTSD had a significant positive effect on believing fake news (β = 0.444, p < .001), PTSD had a statistically significant positive effect on FOMO (β = 0.347, p < .001), and that PTSD and FOMO together had a significant positive effect on believing fake news (β = 0.373, p < .001). Our mediation model was therefore initially validated. Table 3 presents the results of this analysis.

**Table 3:** Regression analysis

Variables	M1: Fake News Belief			M2: FOMO			M3: Fake News Belief		
	B	t	β	B	t	β	B	t	β
PTSD	0.101	9.332^***^	0.444	0.324	6.954^***^	0.347	0.071	6.733^***^	0.315
FOMO							0.090	7.967^***^	0.373
F	84.093^***^			48.359^***^			82.973^***^		
R^2^	0.197			0.120			0.320		

Note: B is the unstandardised coefficient, β is the standardised coefficient; ***p < .001, two-tailed.

Bootstrapped simulations of the mediated model were run 5,000 times using Model 4 of the PROCESS plugin [41]. As shown in Table 4, results demonstrated that PTSD had a significant indirect effect on believing fake news via FOMO (effect = 0.029, boot SE = 0.006, boot 95% CI = 0.019, 0.041), supporting the validity of the mediation model. PTSD also had a significant direct effect on believing fake news (effect = 0.071, boot SE = 0.011, boot 95% CI = 0.005, 0.093).

**Table 4:** Bootstrap test with mediator model

Pathway	Effect	Bootstrap			Standardisation effect
		SE	LLCI	ULCI	
Direct effects	0.071	0.011	0.050	0.093	0.315
Indirect effects	0.029	0.006	0.019	0.041	0.129
Total effect	0.101	0.011	0.080	0.122	0.444

### Moderator analysis

**Fig. 2:**
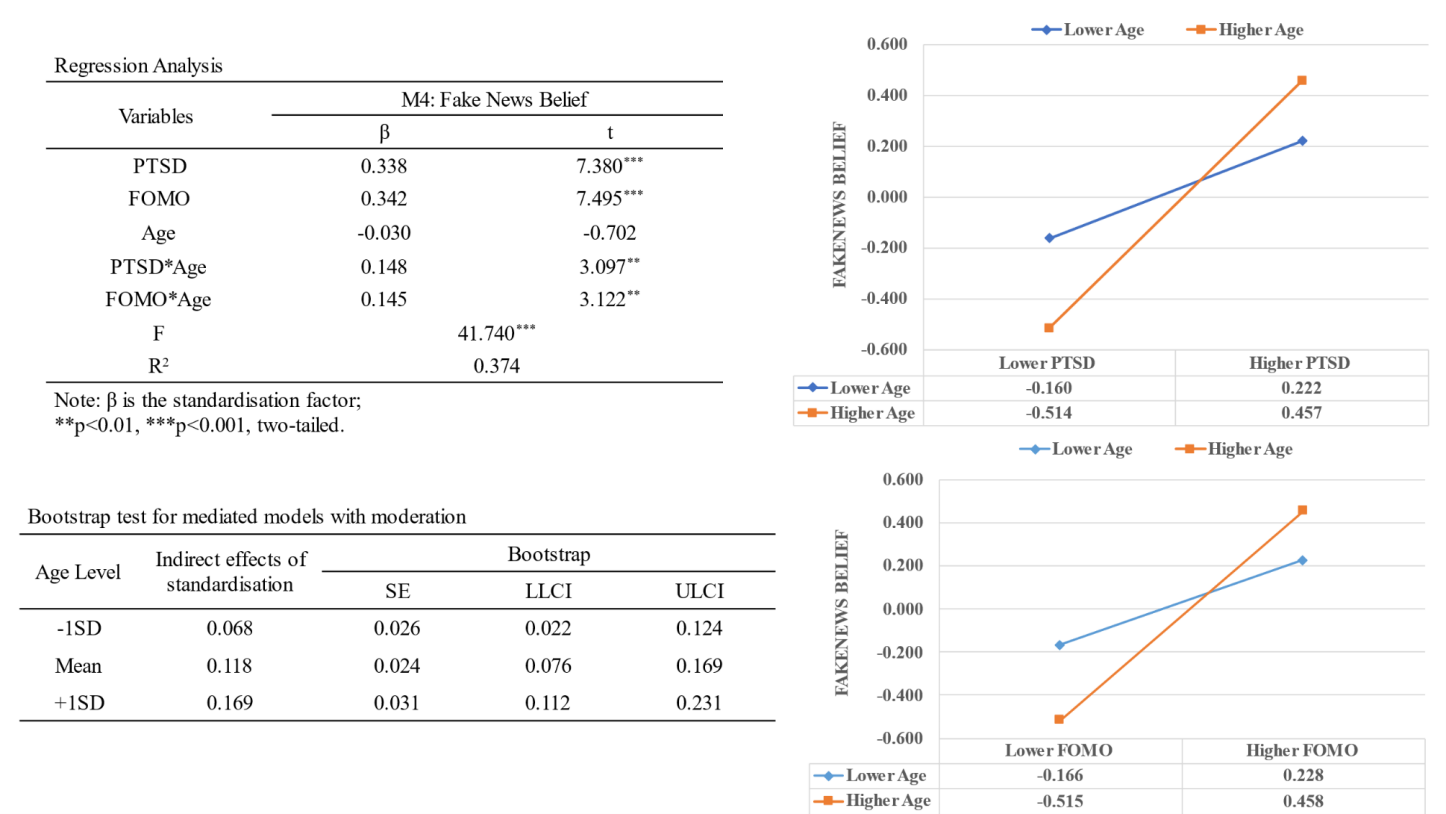
Analysis of the moderating effect of age

We tested age as a possible moderator of the PTSD → Fake News Belief and FOMO → believing fake news pathways. All variables were transformed for normalisation and analysed using Model 15 of PROCESS. Results are shown in Fig. 2.

Analysis revealed a significant positive moderating effect of age on the PTSD → believing fake news pathway (β = 0.148, t = 3.097, p = .002). The positive effect of PTSD on believing fake news was greater when age was higher (+ 1SD) (β = 0.486, t = 6.796, p < .001) and smaller when age was lower (-1SD) (β = 0.191, t = 3.164, p = .002). Age also had a significant positive moderating effect on the FOMO → believing fake news pathway (β = 0.145, t = 3.122, p = .002). The effect of PTSD on FOMO was greater when age was higher (+ 1SD) (β = 0.486, t = 7.870, p < .001) than when and smaller when age was lower (-1SD) (β = 0.197, t = 2.892, p = .004). In addition, bootstrapped results of moderated mediation analysis revealed that the indirect effect was stronger when age was higher.

**Fig. 3:**
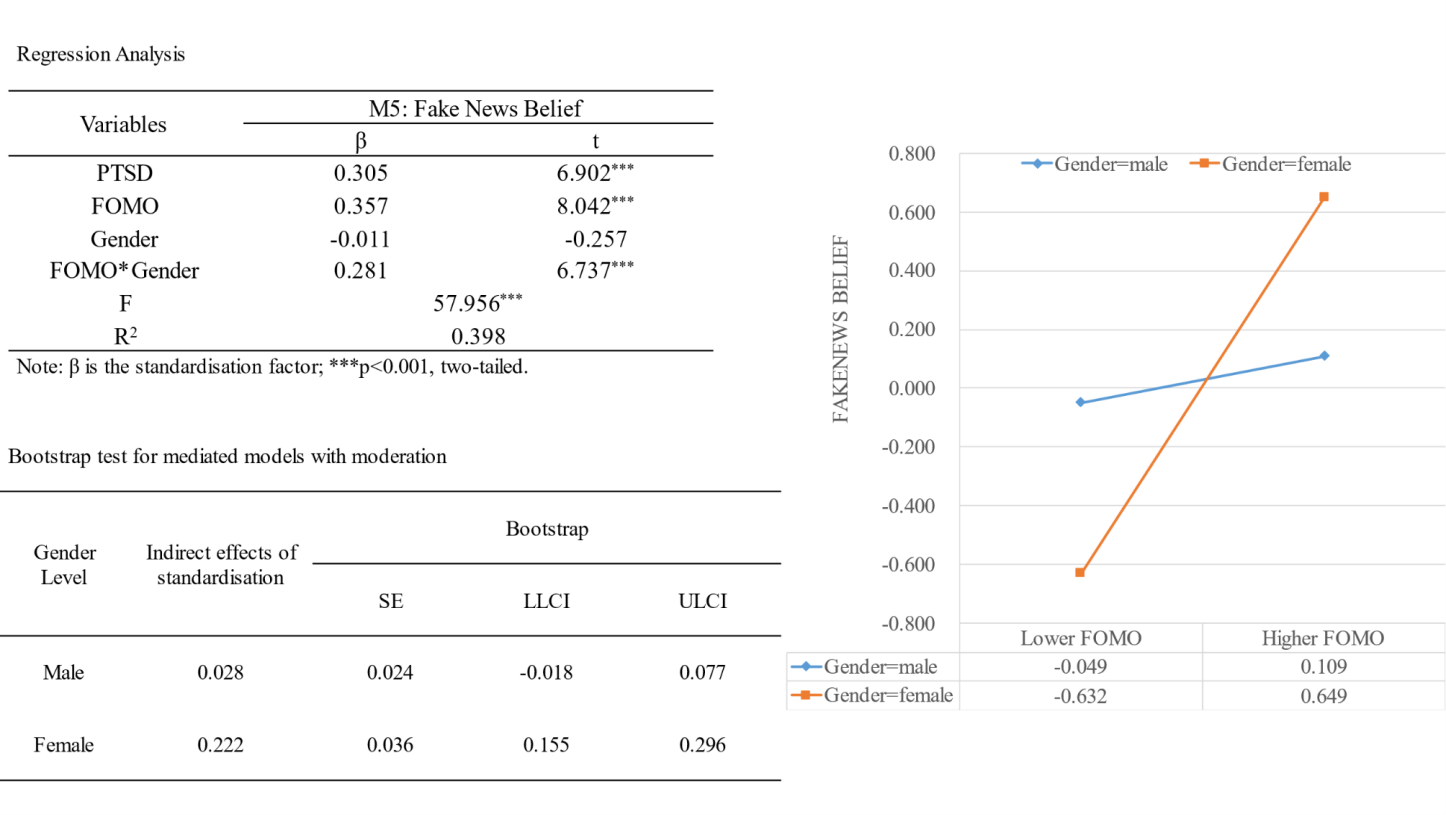
Analysis of the moderating effect of gender

We next tested whether gender moderated the FOMO → believing fake news pathway using the same method as above. Results are shown in Fig. 3.

Gender had a significant positive moderating effect on the FOMO → believing fake news pathway (β = 0.281, t = 6.737, p < .001). Results of a simple slope analysis demonstrated that FOMO had a substantial positive effect on believing fake news in women (β = 0.640, t = 10.754, p < .001) but no such effect in males (β = 0.079, t = 1.279, p = .202). Additional bootstrapped results of the moderated mediation analysis showed that the mediation model was not valid for male participants (effect = 0.028, boot SE = 0.024, boot 95% CI = − 0.018, 0.077). For females, the model was significant (effect = 0.222, boot SE = 0.036, boot 95% CI = 0.155, 0.296).

**Fig. 4:**
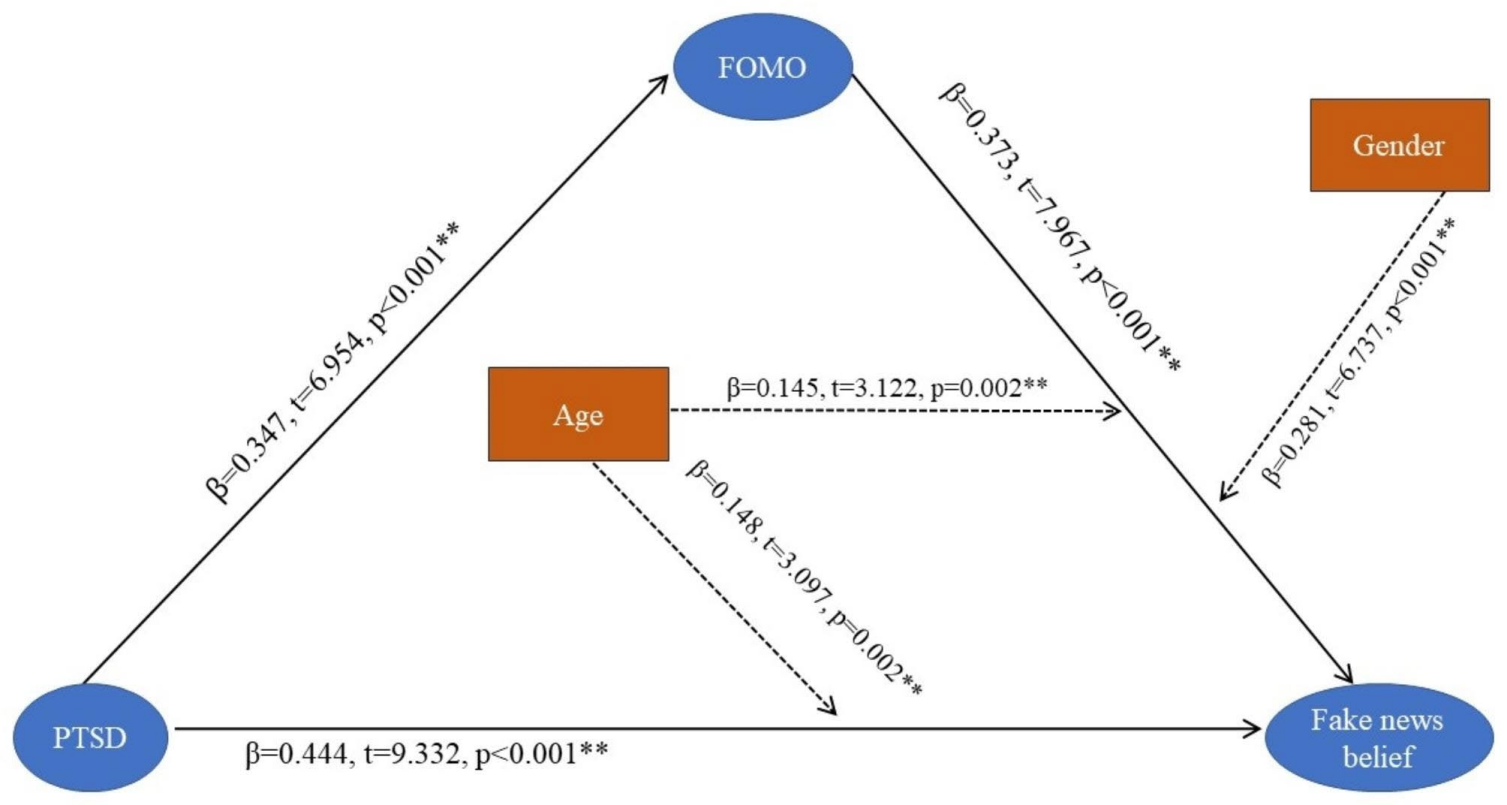
Mediation model with standardized path coefficients

Figure 4 shows the final evaluation of the mediated model with moderating effects.

## Discussion

The primary aim of this study was to examine associations between PTSD, FOMO, and belief of fake news in a sample of earthquake survivors in Wenchuan, China. Results indicated that FOMO was positively associated with PTSD and believing fake news, supporting our hypotheses H1 and H2. This is also consistent with Elhai et al. [7, 8, 27, 28]’s research, while he suggests that FOMO may be an important variable in explaining certain types of psychopathology. We found that the PTSD of Wenchuan earthquake survivors is indeed related to their belief in fake news, supporting H3. This is consistent with findings from Yang et al. [4] who studied a sample of adolescents that had experienced a major earthquake. Next, we found that FOMO mediated the association between PTSD and believing fake news, supporting our hypothesis H4.

Considering the moderating effect of age, we found that it contributed to both direct and indirect effects in the model, with effects being stronger as age increased. This supports our hypotheses H5 and H6 and follows findings from Sela et al. [6], who found moderate associations between depression, FOMO, and problematic Internet use in adolescents. The finding may be explained by younger people typically accessing news and information through a range of friendship groups and social platforms [22], whereas older people tend to access more limited sources of information and may be less able to identify misinformation. Enhancing social skills [6], improving social support networks [4], and using positive thinking therapy [16] may help to ameliorate the negative effects of trust in fake news in those who experience FOMO, particularly older individuals.

We explored age and gender as potential moderating variables of our mediational model following Yang’s [4] approach of treating gender and age as covariates to avoid multicollinearity. Supporting our hypothesis H7, we found that gender moderated the FOMO → believing fake news pathway, such that the effect only existed in females. This is in line with findings from previous studies [16, 30]. It has been reported that women spend more time on social media, perhaps because women use smart devices more for communication whereas men use them more for entertainment such as playing games, a possible reason why women are more likely to experience FOMO [30]. Women are also more likely than men to imagine negative scenarios resulting from natural disasters and public safety emergencies, perhaps due to higher levels of ruminative thinking [13] or higher proneness to stress and anxiety [33].

### Limitations

Our study had several limitations. The sample was limited to a single country, meaning findings may not translate to other locations or cultural contexts. We therefore encourage other researchers to employ a similar research design in other populations to validate our findings. The study relied on self-reported measures to assess the variables of interest. While this follows similar studies [4, 8, 15, 16], we note that more objective measures such as structured diagnostic interviews might strengthen the validity of the findings [15].

### Implications

Despite these limitations, our findings have various theoretical and practical implications. Regarding the former, this is possibly the first study to explore links between PTSD, FOMO, and belief in fake news, and so it makes a significant academic contribution to the evidence base. It also does this in the context of a developing region. The study and its findings are timely due to the substantial increase in the dissemination of fake news during the COVID-19 pandemic.

Regarding practical implications, findings may be useful for policymakers and scholars. Given the observation that females and older individuals are more susceptible to fake news, it may be worth considering the implementation of safeguarding policies for specific demographic subgroups. One potential approach might involve monitoring the use duration of specific applications and issuing tailored security notifications to certain user groups. We also highly recommend the implementation of campaigns to raise awareness of fake news and promote media literacy and fact-checking. The media should assume a more proactive role in fostering awareness about fake news and its consequences. An example that could be followed is goviralgame.com, a UK platform in which players learn to resist manipulation techniques used to spread misinformation about COVID-19 [42]. Relatedly, Chinese media, educational institutions, and government should act responsibly in content creation and management. and engage in multisectoral action to treat those affected by traumatic events.

To mitigate the negative effects of fake news on those suffering from PTSD, future research could explore applicable psychological therapies such as positive thinking therapy [16]. Researchers could also further examine correlations between individual differences such as personality traits and specific platform characteristics to better understand who is most vulnerable to fake news [42].

## Conclusion

This study has shown that PTSD symptoms in Wenchuan earthquake survivors are associated with the FOMO and believing fake news. Additionally, FOMO was found to mediate the association between PTSD and believing fake news. Moderating effects of age on both direct and indirect pathways were observed, and we found that the mediating effect was present among females but not males. Results provide an important reference for those wishing to analyse psychological variables in studies of Internet and social media use in those with PTSD.

## Data Availability

The raw data can be found in https://osf.io/3v9t8/. More details of this study are available from the corresponding author(cgong22@m.fudan.edu.cn), upon reasonable request.
